# Tunneling nanotubes provide a new route for bovine viral diarrhea virus spreading

**DOI:** 10.3389/fvets.2025.1667394

**Published:** 2025-10-10

**Authors:** Jiying Yin, Zehui Zhou, Ning He, Hongming Zhou, Xiaoqun Liu, Yixing Zhao, Longge Zhao, Jiating Zhang, Yanan Zhu, Ying Zong, Naichao Diao, Kun Shi, Rui Du

**Affiliations:** ^1^College of Animal Science and Technology, Jilin Agricultural University, Changchun, Jilin, China; ^2^College of Chinese Medicinal Materials, Jilin Agricultural University, Changchun, Jilin, China; ^3^Laboratory of Production and Product Application of Sika Deer of Jilin Province, Jilin Agricultural University, Changchun, China; ^4^Key Laboratory of Animal Production, Product Quality and Security, Ministry of Education, Jilin Agricultural University, Changchun, China

**Keywords:** bovine viral diarrhea virus, tunneling nanotubes, F-actin, celler transmission, immune evasion

## Abstract

**Introduction:**

Bovine viral diarrhea virus (BVDV) is one of the major pathogens currently endangering the world's cattle industry. It poses serious difficulties in prevention and treatment because it can infect cattle of all ages and the specific mechanism of its cell-to-cell transmission has not yet been fully clarified. Tunneling nanotubes (TNTs) are F-actin-rich tubules that connect to the cytoplasm of nearby cells. They have been found to play an important role in the transmission of several viruses, but studies on BVDV in TNTs have not been reported.

**Methods:**

Firstly, the transwell assay was employed to investigate the transmission routes of BVDV and its capacity to propagate via intercellular junctional structures in the presence of neutralizing antibodies. Secondly, preliminary characterization of these junctional structures was conducted through pharmacological intervention experiments using the microtubule stabilizer paclitaxel, the microtubule disruptor nocodazole, the F-actin disruptors cyclosporine D and spongiosin A, and the gap junction blocker glycine. Subsequently, we validated the composition, spatial positioning, microscopic morphology, and generation characteristics of intercellular junctional structures following BVDV infection. Finally, iSTORM and live-cell fluorescence dynamic imaging techniques, we observed the transmission of BVDV viral particles through TNTs.

**Results:**

Transwell assays demonstrated that BVDV can be transmitted via direct intercellular contact, a mode of transmission unaffected by neutralizing antibodies. Pharmacological studies revealed that only the F-actin disruptors spongin A and cell relaxin D inhibited the formation of this structure, preliminarily identifying it as a tunnel nanotube. Validation experiments confirmed that the composition, spatial orientation, microstructure, and formation direction of this connecting structure align with tunneling nanotube characteristics, further substantiating its identity as TNTs. iSTORM and live-cell fluorescence dynamic imaging revealed that BVDV particles can traverse TNTs to complete intercellular infection.

**Discussion:**

We first report that BVDV can induce the formation of tunneling nanotubes and exploits this route to spread to uninfected cells. Our data highlight a previously unknown route of BVDV spreading, which could have significant implications for celler transmission and immune evasion.

## 1 Introduction

Bovine viral diarrhea virus (BVDV) belongs to the family Flaviviridae, the genus Plague Virus, has a vesicular structure with a diameter of 40–60 nm, and can infect a range of mammals, particularly those belonging to the genus Bovine (1). Infected animals experience a significant decline in production performance, characterized by diarrhea and respiratory symptoms, along with reproductive and immune dysfunctions. These factors pose a serious threat to global animal husbandry (2–4) and result in substantial economic losses for the industry (5). BVDV is known for its ability to evade host immune surveillance and for its vertical transmission through the placenta, leading to persistent infections in offspring (6, 7). This persistence is closely linked to direct cell-to-cell transmission, which serves as the primary route of infection (8). Consequently, understanding the mechanisms of BVDV transmission from cell-to-cell and its role in persistent infection is a pressing scientific inquiry that warrants further investigation.

Tunnel nanotubes (TNTs) are considered to be an important pathway for cell-to-cell transmission of various viruses. Although tunnel nanotubes have been proven to be an important pathway for intercellular transmission of a variety of viruses in recent years (9–11), whether BVDV can be transmitted through this pathway has not yet been reported. Tunnel nanotubes, first discovered in 2004, are filamentous actin-rich tubular structures connected to the cytoplasm of neighboring or distant cells (12). Unlike other cell protrusions, its open ends make intercellular communication easier. The substance has connectivity, thereby mediating efficient communication between cells. TNTs can mediate the transmission of a variety of pathogens in the early stages of its production and has been widely reported as a viral intercellular transmission pathway revealed in recent years. Viruses using TNTs for delivery can protect viral components from the extracellular environment. For example, the formation of TNTs facilitates the delivery of bovine herpesvirus type 1 (BoHV-1) between epithelial cells and fibroblasts (13). The transmission speed of viruses through tunneling nanotubes (TNTs) is significantly faster than that of extracellular transfer, suggesting that direct cell-to-cell transmission is a more effective method for infecting the host compared to extracellular means (14, 15). Furthermore, the intercellular delivery of TNTs by viruses has a high probability of evading the host immune response. For instance, research on the human T-cell leukemia type 1 virus (HTLV-1) indicates that TNTs delivery enables the virus to circumvent host immune surveillance, thereby offering new insights into the mechanisms of viral evasion (16). The presence of a large number of TNTs connections between T cells in HIV-infected individuals provides a new pathway for cell-to-cell transmission of HIV, along which HIV can infect normal cells and is capable of efficient cell-to-cell transmission via TNTs in the presence of neutralizing antibodies, thus evading attack by neutralizing antibodies (17). Djurkovic et al. (18) found that Ebola virus uses tunneling nanotubes as an alternative route of transmission to evade surveillance by the immune system, suggesting a new mode of transmission of the virus within the host. Pepe et al. found that tunneling nanotubes provide a new pathway for the transmission of SARS-CoV-2. The lack of ACE2 receptors in brain cells prevents SARS-CoV-2 from directly infecting the brain, but SARS-CoV-2 can use TNTs to spread from susceptible cells to brain cells to cause brain infection, thus allowing the virus to accelerate viral spread and evade immune surveillance, it suggests a previously unknown mechanism for the spread of SARS-CoV-2, which will likely be used as a way to invade non permissive cells and enhance the pathway of permissive cell infection (11).

We contend that studying the mechanisms of BVDV transmission via TNTs between target cells is likely to lead to effective strategies for the prevention and treatment of BVDV infections in the future.

## 2 Materials and methods

### 2.1 Cells and virus

Madin-Darby Bovine Kidney (MDBK) cells (Bio-68289) was provided by Cell Bank Type Culture Collection of Chinese Academy of Sciences (Cell Bank of TCCCAS, Shanghai, China), and were cultured in Dulbecco's modified Eagle's medium (DMEM; Gibco, Grand Island, NY, USA) supplemented with 10% fetal bovine serum (Gibco) in a humidified incubator at 37 °C with 5% CO_2_. MDBK cells were treated with paclitaxel (10 nM), Nocodazole (50 ng/ml), Cytochalasin D (50 μg/ml), Latrunculin A (10 nM), and Carbenoxolone (1 mM), respectively. BVDV strain NADL (ATCC VR-534) was provided by American Type Culture Collection (ATCC).

### 2.2 Antibodies and reagents

The Bovine Viral Diarrhea Virus Type 1&2 (BVDV-1&2) MAb E2 gp53 was obtained from Veterinary Medical Research & Development; Goat anti-mouse IgG (H+L)-FITC-conjugated (# ab6785) was from abcam; The antibodies anti-F-actin (bs-1571R) were provided by Bioss. TRITC Phalloidin (40734ES75), Cytochalasin D (53215ES03), Paclitaxel (53530ES10) and Nocodazole (51301ES08) were from YEASEN; Latrunculin A (76343-93-6) was provided by Shanghai yuanye Bio-Technology; Carbenoxolone (5697-56-3) was provided by MACKLIN; DiO (C1038) and DiL (C1036) were provided by Beyotime.

### 2.3 Immunofluorescence

MDBK cells were seeded on glass coverslips at a density of 2 × 10^5^ cells/well in 48-well plates and infected at an MOI of 1. At the indicated times after infection, cells were thoroughly washed and fixed with 4% paraformaldehyde (PFA) for 10 min, permeabilized with 0.5% Triton × 100/PBS for 8 min. Then, the plates were blocked in 3% BSA and incubated overnight with anti-BVDV Mab E2 (1:100, # 348, Veterinary Medical Research & Development, USA). Subsequently, the plates were incubated for 2h with Goat Anti-Mouse IgG (H&L)-FITC (1:40, # ab6785, abcam, UK). Cell nuclei were stained with DAPI (4=,6-diamidino-2-phenylindole), and coverslips were mounted onto glass slides by use of FluoroGuard antifade reagent (Invitrogen).

Then, confocal microscope (Leica, STELLARIS5, Germany) was used for observation and image acquisition.

### 2.4 Transwell coculture assay

It has been reported that cell-to-cell transmission of BVDV depends on direct cell-to-cell contact. However, the exact mode of contact is not yet know. In order to gain insight into the mechanisms of BVDV transmission in the presence of neutralizing serum, we used a transwell co-culture system to physically separate infected production cells from target cells. Two different pore sizes of transwell polycarbonate membrane inserts (Corning) with 0.4 and 12 μm pore sizes were used, respectively, of which only the 12 μm pore size allowed the passage of MDBK cells. We inoculated BVDV-infected cells in the upper layer and cultured them using medium with neutralized serum (1:400), and cultured uninfected cells at the bottom of the wells. Concurrently, we established a virus infection group without neutralizing antibodies as a positive control. After 24 h of incubation at 37 °C, cells were fixed and processed for visualization by confocal microscope (Leica, STELLARIS5, Germany).

### 2.5 Scanning electron microscope

In order to observe the morphological characteristics of intercellular TNTs more intuitively, the use of with a scanning electron microscope (SEM can be observed. The cells infected with BVDV were discarded from the culture medium, washed three times with PBS for 5 min each time, discarded the liquid, fixed with 2.5% glutaraldehyde at 4 degrees for 24 h, poured off the fixative, rinsed the samples three times with PBS for 15 min each time, fixed the samples with 1% osmium solution for 1–2 h, carefully removed the osmium waste solution, rinsed it three times for 15 min each time. The samples were fixed with an ethanol solution of gradient concentrations (including 30, 50, 70, 80, 90, and 95% concentrations) for 1–2 h. 70, 80, 90, and 95% of ethanol solution for 15min each, then treated with 100% ethanol for 20 min, and finally, replaced with new 100% ethanol, the sample was placed in 100% ethanol. After that, the samples were dried in a critical point dryer, fixed in the sample stage using a carbon-conducting electrocarbon adhesive, and sputtered by an ion sputtering apparatus for about Pt 120 s, and the images were collected and analyzed using SEM (Hitachi, SU3800, Japan).

### 2.6 Determination of TNTs generation direction

TNTs were stained using DiO and DiL, respectively. One group of colored cells was treated with BVDV (infected cells), then trypsinised and washed, and then co-cultured with another group of normally cultured colored cells (uninfected cells) at 1:1 ratio for 24 h. By doing this, we obtained co-cultures containing both green (DiL) and red (DiO) cells and could observe TNTs development between two colored cell groups.

### 2.7 iSTORM

BVDV-infected cells were discarded from the culture medium and washed with 1 ml PBS once at room temperature (RT). Fix with 0.5 ml fixation solution (3% paraformaldehyde, 0.1% glutaraldehyde in PBS) for 10 min at RT. Wash with 1 ml three times, add 0.5 ml of 0.1 M glycine, and incubate for 7 min. Wash three times, shaking gently for 5 min each wash. Block cells within 200 μl blocking buffer (3% w/v normal goat serum, 0.2% v/v Triton X-100 in PBS) for 15 min. Incubate with anti-BVDV Mab E2 (1:100 dilution) and rabbit anti-F-actin (1:50 dilution) in 100 μl blocking buffer per coverslip at RT for 30 min. Wash with 1 ml PBS three times, rocking gently for 5 min each wash. Incubate with CF 568 Goat Anti-Mouse IgG (H+L) Highly Cross-Adsorbed Secondary Antibody (1:200 dilution) and Alexa Fluor^®^ 647 Goat Anti-rabbit IgG (H+L) Antibody (1:400 dilution) in 100 μl blocking buffer per coverslip at RT for 40 min (19). Detection with iSTORM (INVIEW, 3CM, China).

### 2.8 Live cell fluorescence dynamic imaging system

In order to achieve tracing of BVDV in MDBK cells and to observe more visually whether virus particles can be transmitted via TNTs, the final concentration of 250 μM fluorescent dye DiL was used to incubate with purified BVDV at room temperature for 90 min, and the unbound DiL dye was removed using a NAP-10 column, i.e., fluorescence-labeled BVDV (DiL-BVDV), and the MDBK cells were were inoculated in 12-well cell culture plates at a density of 2.2 × 105 cells/ml, DiL-BVDV was incubated at 37 °C for 1 h, the extracellular unadsorbed viral particles were washed away by PBS, and the live cell membrane nanotubes were stained by DiO for 30 min, after which the culture was replaced with fresh complete medium and continued for 24 h. The nuclei of the cells were stained using the Hoechst 33342 Live Cell Staining Solution The nuclei were stained with Hoechst 33342 Live Cell Staining Solution for 20 min, and images were acquired every 30 min using the Live Cell Real-time Dynamic Fluorescence Imaging System to observe the propagation of intracellular BVDV.

### 2.9 Statistical analysis

All data are expressed as the mean ± standard deviation (mean ± S.E.), which was determined using one-way analysis of variance (ANOVA) and Bonferroni's *post hoc* test. The statistical data are shown as graphs generated using GraphPad Prism 8.0.2 software (GraphPad Software Inc., San Diego, CA, USA). In the figures, ^*^ represents *P* < 0.05, ^**^ represents *P* < 0.01, and ^***^ represents *P* < 0.001.

## 3 Results

### 3.1 BVDV spread relies on direct cell-to-cell contact

**Figure 1:**
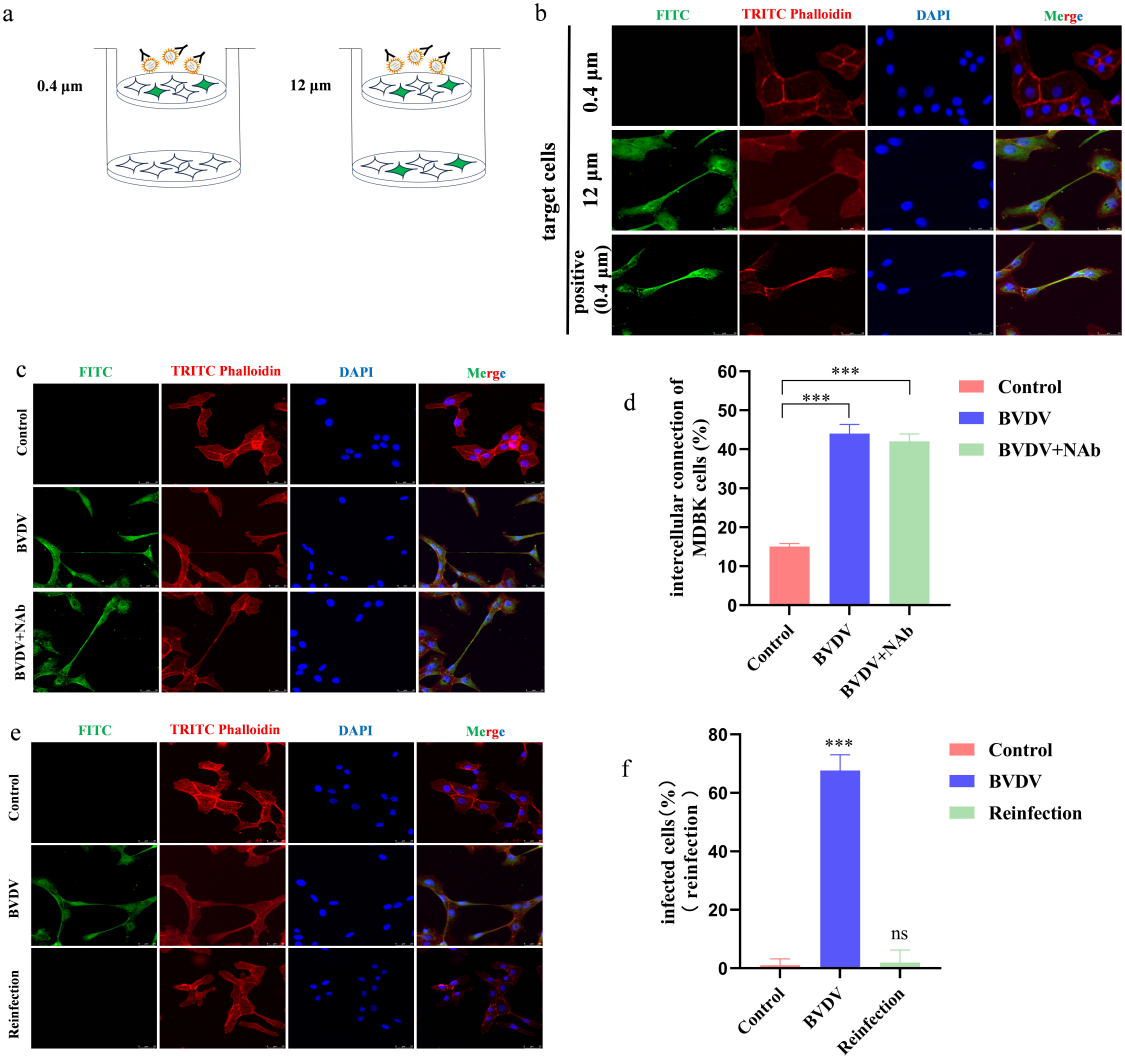
Direct contact between cells is the main way of BVDV spread. **(a)** Schematic representation of the transwell assay. **(b)** In the presence of neutralizing antibodies, MDBK cells can produce TNTs to allow BVDV transmission. **(c)** BVDV can be transmitted in the presence of neutralizing antibodies. **(d)** Quantification of spreading. Number of intercellular connection of MDBK cells were counted in 100 cells in three independent preparations (*n* = 3). Data represented mean ± S.E., ***P < 0.001 compared with control groups. **(e)** Fresh MDBK cells were re-infected by collecting supernatants from virus-infected medium containing neutralizing antibodies. **(f)** Quantification of re-infection of the supernatant (*n* = 3). Data represented mean±S.E., ***P < 0.001 compared with control groups.

We cultured the infected cells on 12μm as well as 0.4 μm cell culture dishes and the extracellular viruses were neutralized by Bovine Viral Diarrhea Virus Type 1 and 2 (BVDV-1&2) Mab, respectively (Figure 1a). The 12 μm pore size allowed the MDBK cells to shuttle freely, while the 0.4 μm pore size only allowed the BVDV viral particles to pass through and did not allow the cells to pass through and the uninfected cells were plated at the bottom of the pore, which showed that the target cells in the BVDV-accepting group were infected by the virus particles, and the presence of virus particles was not detected in the group with the addition of neutralizing antibody, whereas when the pore size was changed to 12 μm, the target cells remained infected despite the presence of neutralizing antibody, indicating that, the transmission between BVDV relies on the direct cell-to-cell contact (Figure 1b). After viral infection of a cell has begun, viral transmission can occur in two fundamentally different ways: (i) viral particles can be released into the external environment and diffuse through the extracellular space until they interact with a new host cell, and (ii) the viral particles can remain associated with the infected cell, thus facilitating direct transmission between infected and uninfected cells, which is referred to as direct cell-to-cell transmission. Studies have shown that BVDV is mainly transmitted by direct cell-to-cell transmission, but the exact mode of direct transmission has not been reported. In order to verify whether BVDV can be transmitted between cells in the presence of neutralizing antibodies, we infected cells using the BVDV NADL strain and in the presence of neutralizing antibodies, respectively, and the results showed that even in the presence of neutralizing antibodies, BVDV was still able to be transmitted between cells, and the number of infected cells was not significantly different from that of the control group (Figures 1c, d). This is consistent with the findings of other scholars that direct cell-to-cell contact is the primary mode of BVDV transmission. To verify the neutralizing effect of the neutralizing antibody, we used the virus-infected supernatant to re-infect healthy target cells, and the indirect immunofluorescence results showed that the virus-infected supernatant did not cause infection of the target cells (Figures 1e, f).

### 3.2 TNTs allows BVDV to spread between MDBK cells

**Figure 2:**
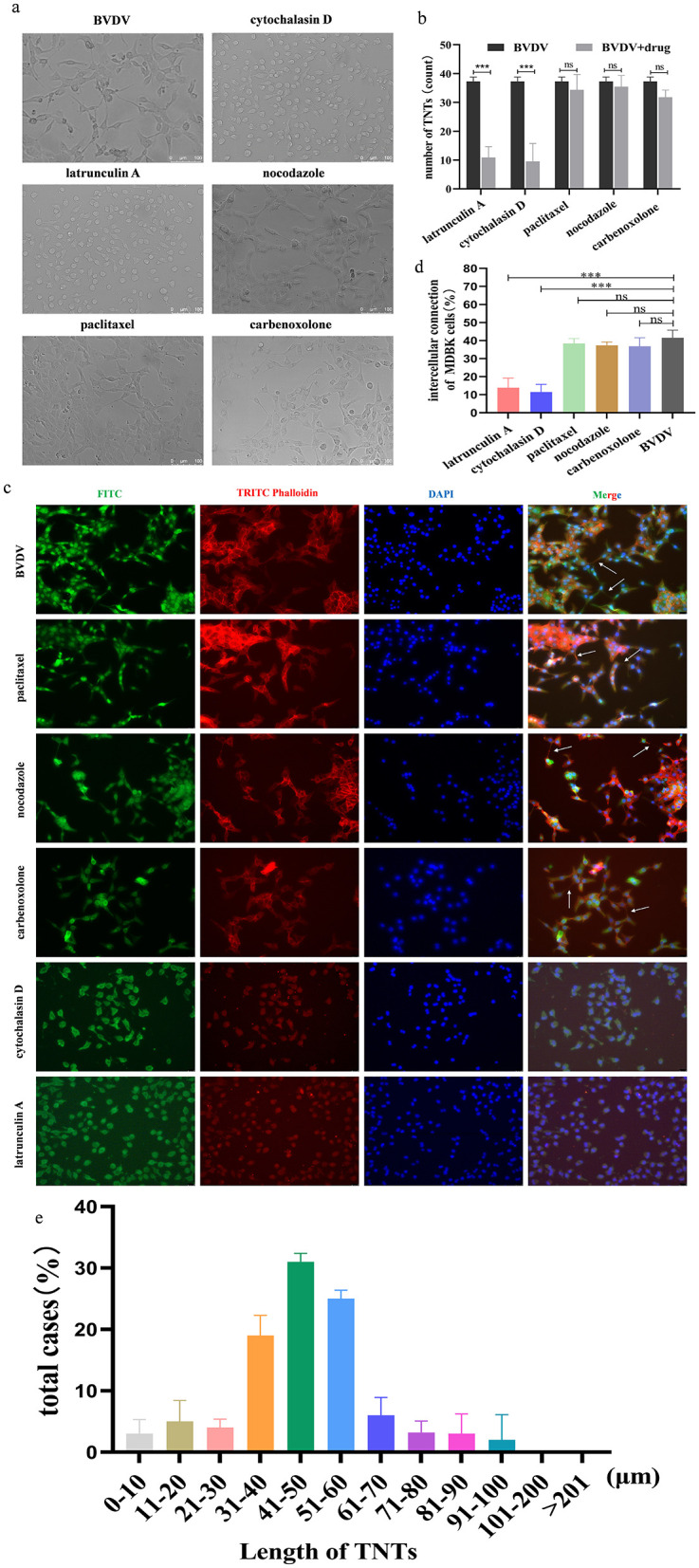
The connecting structures between MDBK cells are TNTs. **(a)** Effect of different drugs on the production of TNTs observed by General Optical Microscope. **(b)** Number of TNTs generation (%). Number of TNTs were counted in 100 cells in three independent preparations (*n* = 3). Data represented mean ± S.E., ***P < 0.001 compared with control groups. **(c)** Effect of different drugs on the production of TNTs observed by indirect immunofluorescence. **(d)** Quantification of TNTs generation (*n* = 3). Data represented mean ± S.E., ***P < 0.001 compared with control groups. **(e)** Distributions of TNT length (*n* = 100).

The above findings suggest that BVDV can be transmitted through direct cell-to-cell contact. To further define the structure of the junction is TNTs, we treated MDBK cells with paclitaxel (microtubule stabilizer), Nocodazole (microtubule disrupting agent), Cytochalasin D (F-actin disrupting agent), Latrunculin A as well as Carbenoxolone (cell gap junction blocker), respectively. The results show that Carbenoxolone (CBX, 10 mM) did not block TNTs induction in MDBK cells indicating that TNTs were not gap junctions. Adding F-actin-disrupting agent latrunculin A (1 μM) or cytochalasin D (2 μg/ml) blocked TNTs induction, whereas microtubule-disrupting agent nocodazole (10 μg/ml) or microtubule-stabilizing agent paclitaxel (10μM) did not alter TNTs induction (Figures 2a, b). To further validate the above results, BVDV E2 and cytoskeleton were stained separately. Observations were made using confocal microscopy with consistent results (Figures 2c, d). These results further confirm that the structure for viral transmission between cells is TNTs. The distribution of nanotube length were quantified and the majority of TNTs lasted for 31–60 μm long (Figure 2e).

### 3.3 BVDV promotes the generation of tunneling nanotubes between MDBK cells

**Figure 3:**
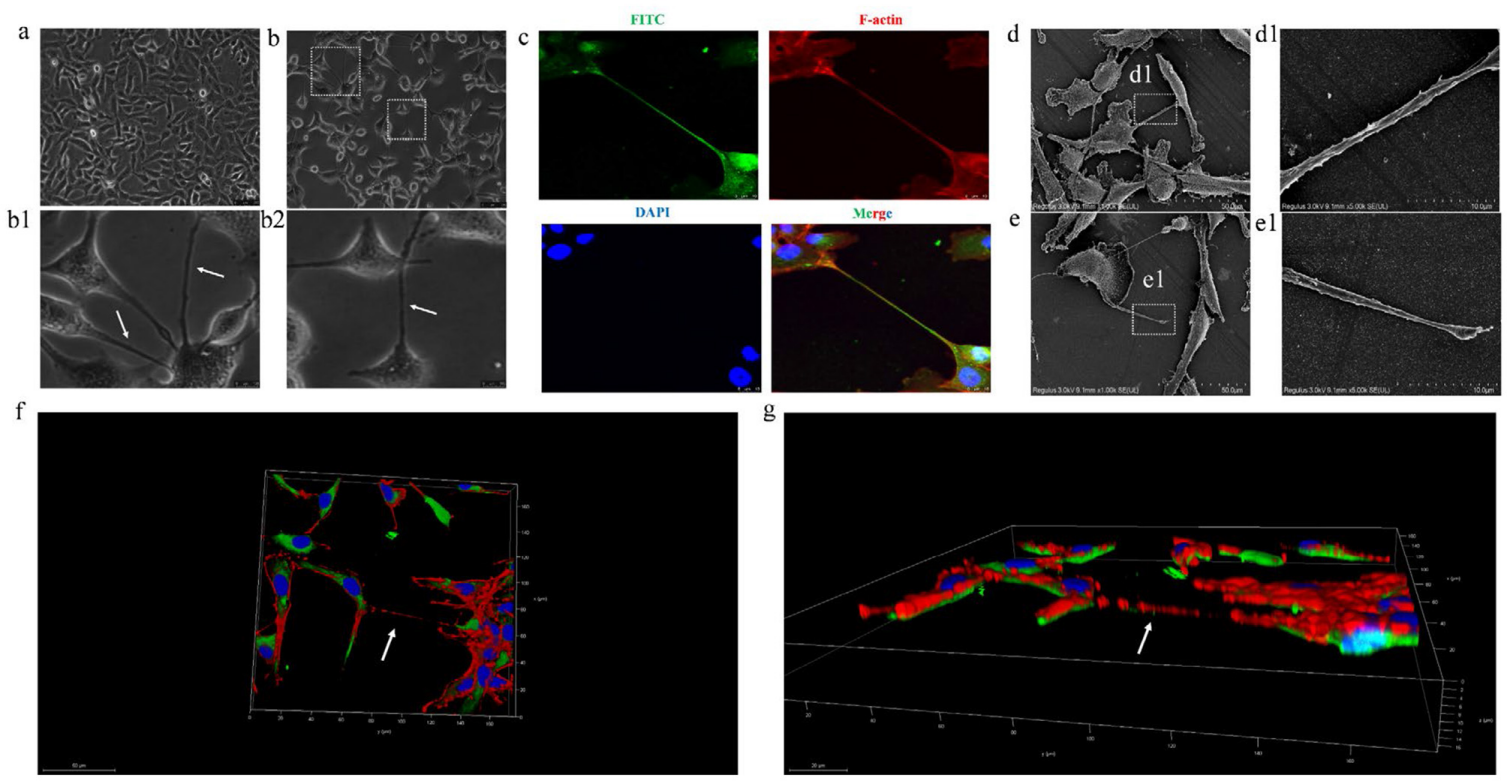
BVDV can promote the production of TNTs between MDBK cells. **(a)** MDBK cells in normal culture. **(b)** TNTs observed with ordinary optical microscope (400×). **(b1, b2)** are high-magnification picture of **(b)**. **(c)** TNTs observed with laser confocal microscopy. **(d)** Scanning electron microscope observation of TNTs micromorphology (open). **(d1)** High-magnification picture of **(d)**. **(e)** Scanning electron microscope observation of TNTs micromorphology (close). **(e1)** High-magnification picture of **(e)**. **(f)** Three-dimensional (3D) reconstruction of TNTs and xy section. **(g)** 3D reconstruction of picture d and xz section.

To verify whether BVDV could promote TNTs generation between MDBK cells, MDBK cells were inoculated with 1 MOI BVDV, and the generation of TNTs between cells was observed using light microscopy. The results showed that BVDV could significantly stimulate the generation of TNTs between MDBK cells (Figures 3a, b, b1, b2). Since a distinctive feature of TNTs is the F-actin-rich membrane structure, in order to further validate the above results, we specifically labeled them with BVDV E2 antibody and F-actin antibody, respectively, and the results showed that the F-actin antibody successfully labeled the junction structure, confirming that the intercellular junctions were indeed TNTs (Figure 3c). We next explored the micromorphology of TNTs using scanning electron microscopy and confirmed that two typical morphological features of TNTs, open structure and blind end structure, could be observed in infected cells, which is consistent with the microtubule morphology of TNTs (Figures 3d, d1, e, e1). Another distinctive feature of TNTs is that they are not in contact with the underlying matrix in which the cells grow, and are a special structure suspended in the extracellular matrix. Finally, We also explored the spatial location of TNTs, and the results showed that TNTs are indeed long-distance cellular junctions in the suspended cell culture medium, rather than being immediately attached to the bottom surface of the cell culture plate, which is consistent with the spatial location of TNTs (Figures 3f, g). In summary, the three aspects of the composition, microscopic morphology, and spatial location of the intercellular connecting structure after BVDV infection were verified, and all of them were consistent with the typical features of TNTs, so the connecting structure was finally identified as tunneling nanotubes.

### 3.4 TNTs tends to be generated from infected cells to uninfected cells

**Figure 4:**
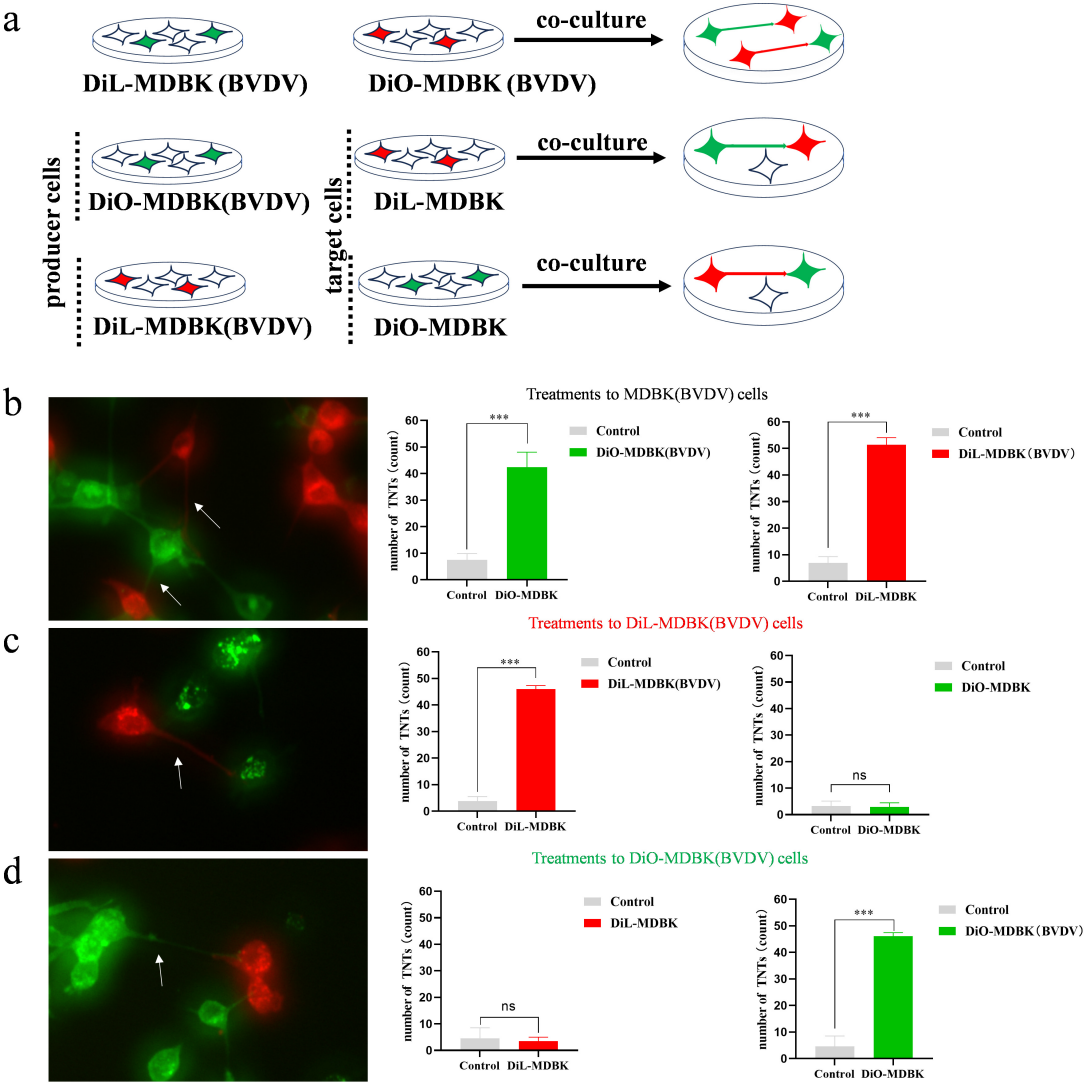
TNTs tend to develop from infected to uninfected cells. **(a)** Experiment procedure for co-culture of infected cells and uninfected cells. **(b)** Both DiO and DiL labeled infected cells (*n* = 3). Data represented mean ± S.E., ***P < 0.001 compared with control groups. **(c)** DiO labeled uninfected cells and DiL labeled infected cells (*n* = 3). Data represented mean ± S.E., ***P < 0.001 compared with control groups. **(d)** DiL labeled uninfected cells and DiO labeled infected cells (*n* = 3). Data represented mean±S.E., ***P < 0.001 compared with control groups.

It has been shown that TNTs is a defense mechanism spontaneously generated by cells in response to dangers that include viral infection. In order to explore the TNTs generation mechanism, MDBK cells were distinguished into two groups. One group was infected with BVDV and then the infected cells were labeled with DIL. And then co-cultured with another color group of uninfected cells labeled with DIO. By doing this, we obtained co-cultures containing both green (DIO) and red (DIL) cells and could observe TNTs development between two colored cell groups (Figure 4a). The results show that both DIO and DIL labeled infected cells, TNTs can both develop from red cells to green cells and develop from green cells to red cells (Figure 4b). When the red cells were are infected by BVDV, there were a significant number of red TNTs developed from the red cells to the green cells, but not from the green cells to the red ones (Figure 4c). To further confirm this hypothesis, we used DIO to label infected cells, DIL to label the uninfected cells, and co-cultured them again. The results showed that the direction of TNTs production all extended from green to red, which is consistent with the results of other studies (Figure 4d). so we concluded that TNTs tends to be produced from infected cells to uninfected cells. This finding is consistent with the characterization of the direction of production of TNTs, and also further confirms that the structures are TNTs. we also propose the reasonable hypothesis that the production of TNTs may be a mode of self-protection when MDBK is infected with BVDV.

### 3.5 iSTORM observation of BVDV within TNTs

**Figure 5:**
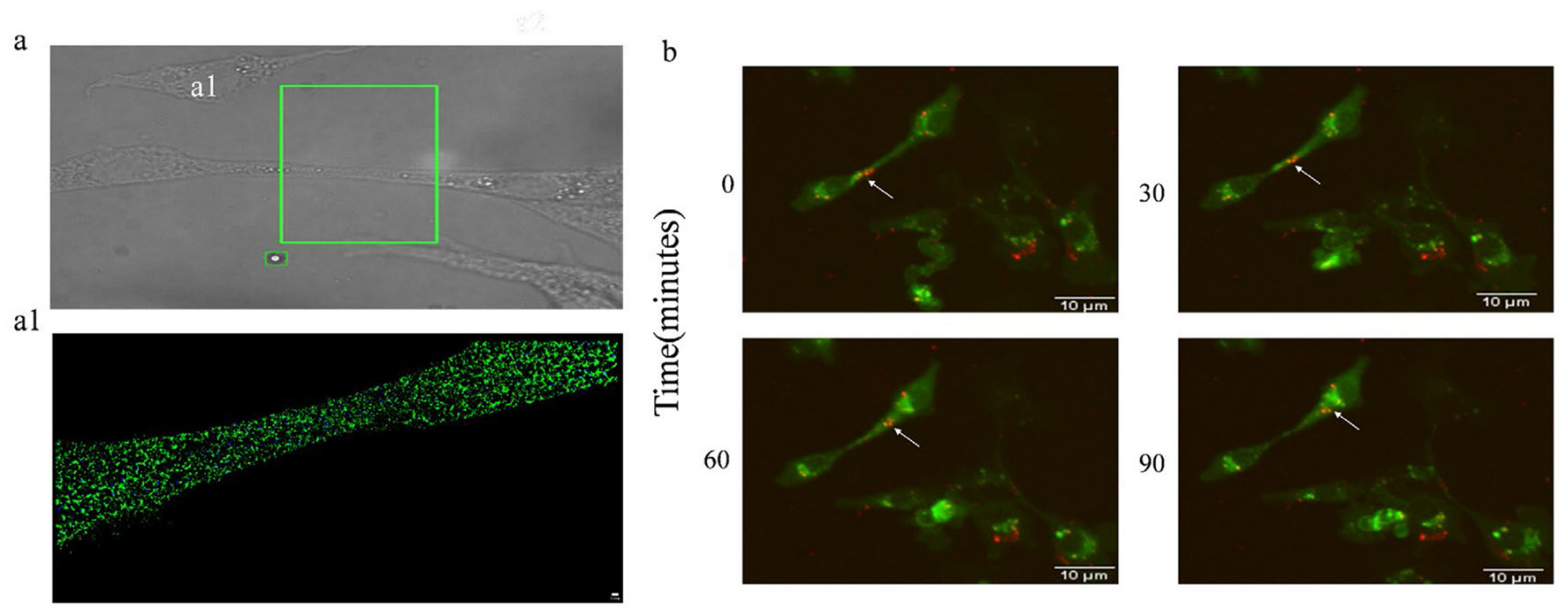
BVDV can be transmitted via TNTs. **(a)** iSTORM observation of BVDV in TNTs. **(a1)** High-magnification picture of **(a)**. **(b)** The Live Cell Dynamic Imaging Analysis System showed that BVDV can be transmitted in TNTs.

We have clearly observed the microstructure of TNTs, but the limited SEM resolution did not observe the presence of viral particles within TNTs. In order to observe whether TNTs are a channel for BVDV transmission, we specifically labeled BVDV and TNTs with BVDV E2 antibody and F-actin antibody, respectively. The presence of viral particles in TNTs was observed using the higher resolution iSTORM, which showed that BVDV (blue) particles in TNTs (green) could be clearly observed, indicating the presence of viral particles within TNTs (Figures 5a, a1). This finding provides direct evidence that TNTs mediate BVDV cell-to-cell transmission.

### 3.6 BVDV can use TNTs for dynamic propagation

The iSTORM results indicated the presence of viral particles in TNTs, in order to further investigate whether the viral particles can be transmitted from cell-to-cell through TNTs, the cells and viruses were labeled using DiL and DiO, respectively, and the live cell fluorescence dynamic imaging system was used to observe in real time whether the viral particles were transmitted through TNTs or not, the results showed that the red-labeled viruses were located in the proximal end of the TNTs connecting two The results showed that the red labeled virus was located at the proximal end of the TNTs connecting the two cells at 0 min of shooting, and with the prolongation of time, the BVDV particle BVDV could be seen to be transferred along the TNTs, and the labeled viral particles had been transferred to the cytoplasm at 90 min of shooting (Figure 5b), which directly confirmed that the TNTs could be used as an effective pathway for the cell-to-cell transmission of viral particles (Supplementary Presentation 1). Simultaneously, we calculated the migration rate of the viral particles to be 187.93 ± 16.99 nm/min.

## 4 Discussion

Bovine viral diarrhea virus (BVDV) is a significant pathogen impacting the global cattle industry, resulting in substantial economic losses for farmers annually. The World Organization for Animal Health (OIE) classifies BVDV as a category B infectious disease, while in China, it is categorized as a category two infectious disease due to its detrimental effects. Despite its prevalence, the precise pathogenic mechanisms of BVDV remain incompletely understood. Currently, BVDV is widespread, having been identified in 88 countries around the world. Consequently, comprehending the transmission dynamics of BVDV is crucial for mitigating its epidemic spread.

Tunneling nanotubes (TNTs) represent a novel mechanism for cell-to-cell communication, first identified by German scientist Hans-Hermann Gerdes in 2004. To date, TNTs have been observed among a diverse array of cell types, including rat adrenal pheochromocytoma PC12 cells (20), human renal epithelial cells HEK293T (21), human renal proximal tubular epithelial cells RPTEC (22), bladder tumor cells RT4 and T24 (23), cervical cancer cells HeLa (24), NK cells (25), human lung cancer cells A549 (26), and acute monocytic leukemia cells THP-1 (27). TNTs can form between different cell types; for instance, endothelial progenitor cells (EPC) co-cultured with human umbilical vein endothelial cells (HUVEC) (28), human renal epithelial cells HEK293T co-cultured with renal fibroblasts COS-7 (29), hematopoietic stem cells HSC co-cultured with macrophages (30), and bladder cancer cells T24 co-cultured with RT4 (31). In the present study, we report for the first time that BVDV induces the production of TNTs between MDBK cells. An additional significant finding in the study of TNTs is their ability to facilitate the cell-to-cell transmission of pathogens, including bacteria, viruses, and prions, which are believed to function as ‘highways' for the transport of materials between cells. Viruses can infect host cells over considerable distances in the extracellular environment, which is the primary mechanism of viral transmission. Recent studies indicate that, in addition to this extracellular route, viruses can also be transmitted between cells via tunneling nanotubes (TNTs) at a significantly faster rate than through extracellular transfer. This finding suggests that direct intercellular transfer may be a more effective method for viral infection than extracellular transmission (32, 33). For instance, the presence of numerous TNTs connecting T cells in HIV-1-infected patients offers a novel pathway for the cell-to-cell transmission of HIV-1, allowing the virus to traverse the outer surface of TNTs to infect adjacent cells (17). Furthermore, a study by Djurkovic et al. (18) revealed that the Ebola virus utilizes tunneling nanotubes as an alternative route for dissemination, proposing a new model for Ebola virus (EBOV) spread within the host. Additionally, research by Pepe demonstrated that tunneling nanotubes facilitate the spread of SARS-CoV-2, highlighting a previously unknown mechanism for the virus's transmission that may enable it to invade non-permissive cells and enhance infection in permissive cells (11). Moreover, the intercellular transmission of viruses via TNTs is likely to evade the host immune response, thereby enhancing viral survival. In the present study, we report for the first time that bovine viral diarrhea virus (BVDV) significantly stimulates the production of TNTs between MDBK cells, providing a compelling explanation for BVDV's ability to evade host immune surveillance.

Cell–cell communication is a fundamental cellular process that occurs through various mechanisms, including microtubules, gap junctions, and tunneling nanotubes (TNTs). To ascertain whether these intercellular junctions are indeed TNTs, we treated cells with Nocodazole, Paclitaxel, CBX, latrunculin A, and cytochalasin D (34). Nocodazole and Paclitaxel serve as microtubule-disrupting and stabilizing agents, respectively, while CBX functions as a blocker of cell gap junctions. Latrunculin A and cytochalasin D are agents that disrupt F-actin. Our findings indicated a significant reduction in the number of TNTs in cells treated with latrunculin A and cytochalasin D. In contrast, the differences in the number of TNTs among the Nocodazole, Paclitaxel, and CBX treated groups were not statistically significant when compared to the control group. This supports the conclusion that these cellular junctional structures are indeed TNTs, rather than microtubules or gap junctions. Meanwhile, we also verified the composition, spatial location, and microscopic morphology of the intercellular connecting structure after BVDV infection, and finally identified the connecting structure as tunneling nanotubes (TNTs). What's more, with the help of super-resolution microscopy (iSTORM) and live-cell fluorescence dynamic imaging, it was confirmed that BVDV virus particles could propagate along the TNTs and complete the transcellular infection.

The formation direction of tunneling nanotubes (TNTs) has been linked to the characteristics of infected cells. TNTs may serve as a self-protection mechanism for cells, facilitating the transfer of cellular material or energy from uninfected cells to infected ones in response to harmful stimuli. Wang successfully induced the formation of TNTs between rat hippocampal astrocytes and neurons by culturing them in hydrogen peroxide (H_2_O_2_) or serum-depleted medium. Their findings indicated that when these two cell types were mixed in culture, uninfected cells consistently directed their growth toward infected cells to form TNTs. In the current study, we used BVDV-infected cells and subsequently labeled the infected cells and co-cultured them with uninfected cells. Our observations revealed that TNTs predominantly extended from infected cells toward uninfected cells. This aligns with findings from other studies that demonstrate TNTs typically extend from infected to uninfected cells. This not only further confirms that the structures are TNTs, but also that this may be a mode of self-protection when MDBK is infected with BVDV. However, the mechanisms underlying the directionality of TNTs formation and their localization to target cells remain unclear.

TNTs provide BVDV with a “stealth” mode of intercellular transmission, enabling it to evade surveillance by the host's humoral immune response (antibodies). This is crucial for the virus to establish and maintain persistent infection (PI) within the host. Currently recognized immune evasion by BVDV primarily occurs through classical mechanisms, such as antigenic variation of the E^rns^ glycoprotein to escape neutralizing antibodies, and suppression of the type I interferon response mediated by the N^pro^ protein (35, 36). Our research proposes a novel complementary mechanism: tunnel nanotubes (TNTs) may provide an “immune-privileged pathway” for intercellular viral transmission. Viral transmission via TNTs occurs entirely at direct cell-cell junctions, circumventing exposure of viral particles to the extracellular space. Consequently, neutralizing antibodies generated within the host fail to recognize and eliminate viruses propagating through this pathway. This provides a compelling explanation for viral persistence in hosts where the immune system has already produced specific antibodies. In this study, we observed abundant TNTs forming between infected cells and neighboring cells, with BVDV capable of exploiting these structures for transmission. Crucially, our *in vitro* experiments demonstrated that viral infection could still propagate from infected cells to neighboring cells under culture conditions containing neutralizing antibodies, providing direct experimental evidence that TNT-mediated transmission evades antibody neutralization. In summary, we propose TNTs as a novel and critical component of BVDV immune evasion. They provide a physically spatial solution for circumventing humoral immunity, significantly enhancing the virus's persistence and transmissibility within the host.

Current BVDV vaccines based on inducing neutralizing antibodies primarily function by clearing extracellular viral particles, thereby blocking their transmission via humoral pathways (37, 38). However, BVDV transmission via TNTs enables the virus to spread “invisibly” between cells, potentially circumventing the humoral immune response induced by vaccines. This may explain why certain vaccines fail to achieve 100% protective efficacy. This suggests that future vaccine design strategies may need to consider simultaneously eliciting robust cellular immune responses to effectively recognize and eliminate cells infected via TNTs. Regarding BVDV eradication strategies, traditional approaches rely on accurately identifying and eliminating persistently infected (PI) animals, which serve as reservoirs for the virus within herds (39). The TNTs mechanism indicates that viral maintenance and dissemination within PI animals may be more “concealed” than previously understood. For instance, the virus may spread between immune cells (such as macrophages) via TNTs, thereby evading host immune clearance more effectively. This underscores the paramount importance of developing highly sensitive detection methods (capable of identifying low-level infections or cell-associated viruses) and rigorously implementing biosecurity measures within eradication programmes. At the population health management level, as TNTs-mediated transmission is faster and more covert than conventional routes, it may be less susceptible to certain disinfectants or physical barriers designed to block extracellular viral transmission. Direct animal-to-animal contact should be minimized and stocking densities optimized, particularly during high-risk periods (peripartum) and in high-risk locations (farrowing units).

**Figure 6:**
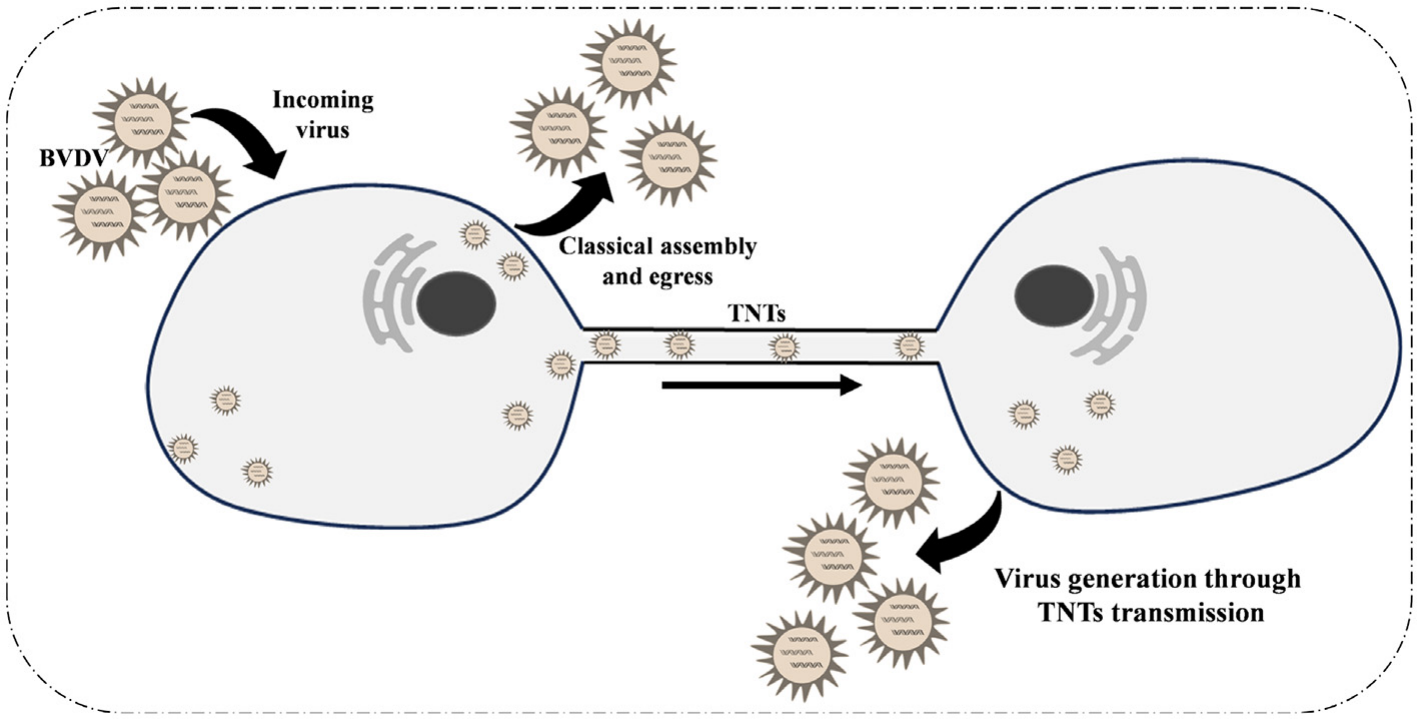
Schematic diagram of the mechanism of tunnel nanotubes as a new pathway for intercellular transmission of BVDV.

In summary, we report for the first time that Bovine Viral Diarrhea Virus (BVDV) infection induces the formation of tunneling nanotubes (TNTs) between MDBK cells (Figure 6). Our findings indicate that BVDV viral particles can propagate through these TNTs structures to infect neighboring cells. Furthermore, the presence of neutralizing antibodies does not impede this transmission pathway, providing a compelling explanation for the immune evasion exhibited by BVDV. This study reveals, for the first time, that TNTs play a crucial role in facilitating BVDV transmission.

## Data Availability

The original contributions presented in the study are included in the article/Supplementary material, further inquiries can be directed to the corresponding authors.
