# Supplementary figures and images for: Tunneling nanotubes provide a new route for bovine viral diarrhea virus spreading
Source: Front Vet Sci. 2025 Oct 10;12:1667394. doi: 10.3389/fvets.2025.1667394 (PMC12550282; doi:10.3389/fvets.2025.1667394)

## Slide 1
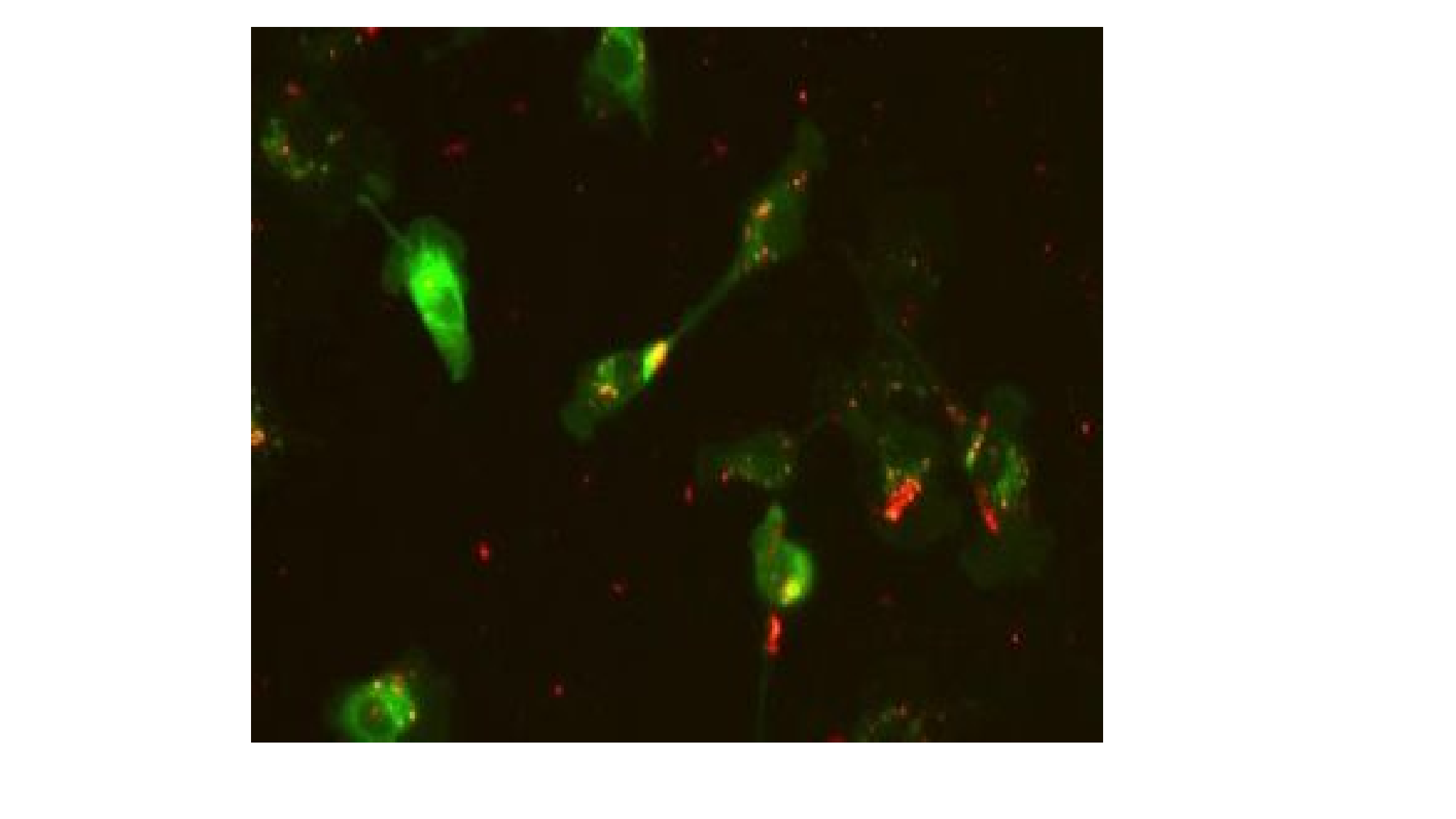

Supplement: Supplementary file 1 [file Presentation_1.PPTX]
